# Reproducibility of an HPLC-ESI-MS/MS Method for the Measurement of Stable-Isotope Enrichment of *in Vivo*-Labeled Muscle ATP Synthase Beta Subunit

**DOI:** 10.1371/journal.pone.0026171

**Published:** 2011-10-12

**Authors:** Sarah Everman, Zhengping Yi, Paul Langlais, Lawrence J. Mandarino, Moulun Luo, Christine Roberts, Christos S. Katsanos

**Affiliations:** 1 Center for Metabolic and Vascular Biology, School of Life Sciences, Arizona State University, Tempe, Arizona, United States of America; 2 Mayo Clinic Arizona, Scottsdale, Arizona, United States of America; 3 Department of Pharmaceutical Sciences, Eugene Applebaum College of Pharmacy/Health Sciences, Wayne State University, Detroit, Michigan, United States of America; University of Helsinki, Finland

## Abstract

We sought to evaluate the reproducibility of a liquid chromatography-tandem mass spectrometry (LC-MS/MS)-based approach to measure the stable-isotope enrichment of *in vivo*-labeled muscle ATP synthase β subunit (β-F1-ATPase), a protein most directly involved in ATP production, and whose abundance is reduced under a variety of circumstances. Muscle was obtained from a rat infused with stable-isotope-labeled leucine. The muscle was homogenized, β-F1-ATPase immunoprecipitated, and the protein was resolved using 1D-SDS PAGE. Following trypsin digestion of the isolated protein, the resultant peptide mixtures were subjected to analysis by HPLC-ESI-MS/MS, which resulted in the detection of multiple β-F1-ATPase peptides. There were three β-F1-ATPase unique peptides with a leucine residue in the amino acid sequence, and which were detected with high intensity relative to other peptides and assigned with >95% probability to β-F1-ATPase. These peptides were specifically targeted for fragmentation to access their stable-isotope enrichment based on MS/MS peak areas calculated from extracted ion chromatographs for selected labeled and unlabeled fragment ions. Results showed best linearity (R^2^ = 0.99) in the detection of MS/MS peak areas for both labeled and unlabeled fragment ions, over a wide range of amounts of injected protein, specifically for the β-F1-ATPase_134-143_ peptide. Measured stable-isotope enrichment was highly reproducible for the β-F1-ATPase_134-143_ peptide (CV = 2.9%). Further, using mixtures of synthetic labeled and unlabeled peptides we determined that there is an excellent linear relationship (R^2^ = 0.99) between measured and predicted enrichment for percent enrichments ranging between 0.009% and 8.185% for the β-F1-ATPase_134-143_ peptide. The described approach provides a reliable approach to measure the stable-isotope enrichment of *in-vivo*-labeled muscle β-F1-ATPase based on the determination of the enrichment of the β-F1-ATPase_134-143_ peptide.

## Introduction

There are well-established approaches to measure the protein synthesis in skeletal muscle using stable-isotope-labeled amino acids [1]. These approaches are based on the *in vivo*-metabolic labeling of proteins followed by the measurement of the stable-isotope enrichment of, typically, a mixture of proteins using gas chromatography-mass spectrometry (GC-MS). Measurement of this enrichment provides the means to determine a global/average rate of synthesis across proteins in skeletal muscle. Measurement of the enrichment of a single protein that is present in small amounts in skeletal muscle cannot, however, be practically performed using these approaches because of the low mass sensitivity of the GC-MS. Yet, it is the measurement of the enrichment at the individual protein level that it is necessary for the determination of the rate of synthesis of a specific protein.

At any time, muscle requires a continuous supply of energy in the form of adenosine triphosphate (ATP), which supports various metabolic processes within muscle and maintains muscle's function. The demand for ATP production in muscle increases several-fold as a result of physical activity. More than 90% of ATP in skeletal muscle is produced by the enzyme ATP synthase in mitochondria. The beta subunit of the ATP synthase (β-F1-ATPase) is important from a functional standpoint because it forms the catalytic site of the enzyme, and thus has a key role in determining the capacity for ATP production. In circumstances associated with reduced capacity for ATP production in muscle, such as obesity [2] and aging [3], reduced amounts of muscle β-F1-ATPase have been reported [4], [5]. Reduced amounts of β-F1-ATPase have also been reported in liver and tumors in conditions associated with hypothyroidism and cancer, respectively [6], [7], [8].

Understanding the mechanisms behind the reduced levels of β-F1-ATPase in skeletal muscle requires knowledge of the rates of synthesis/breakdown of β-F1-ATPase. In addition to maintaining the quantity of muscle β-F1-ATPase, the rate of synthesis of β-F1-ATPase plays a key role in maintaining the quality of protein by continuously renewing the β-F1-ATPase pool in skeletal muscle. As a result of the latter process, older and/or damaged molecules of the protein are replaced by de novo synthesized β-F1-ATPase molecules. A key part in the overall process of determining the kinetics of muscle β-F1-ATPase in association with the infusion of stable-isotope amino acids is the ability to reliably measure the stable isotope enrichment of β-F1-ATPase. Recently, Bateman et al. [9] have described a liquid chromatography-tandem mass spectrometry (LC-MS/MS) approach for the measurement of amyloid-β stable-isotope enrichment from cerebrospinal fluid. However, the applicability of this approach and its reproducibility for the measurement of enrichment of a protein from skeletal muscle, such as the muscle β-F1-ATPase, has not been evaluated. Lower protein abundance and turnover rates, ultimately resulting in lower levels of labeled proteins in skeletal muscle compared to cerebrospinal fluid, as well as differences in the ionization of protein-associated peptides and their fragmentation patterns, all necessitate the need to evaluate the applicability of LC-MS/MS to specifically measure the stable-isotope enrichment of muscle β-F1-ATPase.

The overall goal of this research was, therefore, to determine the reproducibility of an LC-MS/MS method for the measurement of stable-isotope enrichment of *in vivo*-labeled muscle β-F1-ATPase. We specifically sought to determine if there is a specific β-F1-ATPase peptide that can provide reproducible measurements to be used for the determination of β-F1-ATPase enrichment. The following were evaluated for β-F1-ATPase tryptic-peptides following isolation of β-F1-ATPase from rat skeletal muscle: 1) the MS/MS signal linearity in the detection of labeled and unlabeled fragment ions of β-F1-ATPase peptides over a range of injected amounts of protein, 2) the reproducibility of the measured enrichment across β-F1-ATPase peptides, and 3) the linearity in the measured enrichment determined by using mixtures containing various ratios of labeled to unlabeled synthetic peptides for the β-F1-ATPase peptide with the most reproducible measurement of its enrichment.

## Methods

### Ethics statement

The study protocol and procedures were reviewed and approved by the Institutional Animal Care and Use Committee at Arizona State University (Protocol ID 07-939R).

### Selection of the stable-isotope amino acid

L-[2,3,3,4,5,5,5,6,6,6-^2^H_10_]leucine, which is a stable-isotope of the amino acid leucine was selected to label leucine residues in the β-F1-ATPase for several reasons: 1) leucine is abundant in β-F1-ATPase and this increases the chances for detecting leucine-containing peptides that meet our criteria (discussed below); 2) the specific stable-isotope of leucine has a mass that is large enough to separate the isotope envelopes between labeled and unlabeled peptides, allowing for easier identification; 3) although not the focus of the present study, calculation of the rate of synthesis of a protein requires determination of the precursor (i.e. leucine) enrichment, which can be determined in a practical way when using the selected stable-isotope of the amino acid leucine. Specifically, in the selected d10-leucine, and as a result of the transamination reaction, the deuterium attached to the alpha carbon of the amino acid, as is also the case for the hydrogen for endogenous leucine, is rapidly exchanged for hydrogen via alpha-ketoisocaproic acid metabolism *in vivo* resulting in the production of d9-leucine inside the cell [10]. That way, both unlabeled and d9-labeled leucine are introduced intracellularly in the same way before incorporation into the protein, and under these conditions enrichment with labeled leucine that has undergone transamination measured in peripheral blood is the same as that measured in muscle [1], [11], allowing for a convenient and practical determination of the precursor enrichment.

### Animals and infusion protocol

Muscle for the experiments was obtained from two adult male Sprague-Dawley rats (Charles River, Wilmington, MA). Prior to the collection of muscle, d10-leucine was infused (50 mg/kg/hour; Cambridge Isotope Laboratories, Inc., Andover, MA) for seven hours intravenously in one of the rats to increase the incorporation of stable-isotope-labeled leucine into muscle β-F1-ATPase, while the other rat received a comparable rate of infusion of saline.

### Muscle homogenization, β-F1-ATPase immunoprecipitation, SDS-PAGE, and in-gel digestion procedures

Approximately 100 mg of vastus lateralis muscle was homogenized in ice-cold freshly prepared buffer (1 mL/100 mg tissue; 20 mM Hepes pH 7.6, 1 mM EDTA pH 8.0, 250 mM sucrose, 5 mM NaF, 1 mM Na-pyrophosphate, 1 mM ammonium molybdate, 1 mM Na_3_VO_4_, 10 ug/ml aprotinin, 10 ug/ml leupeptin, 250uM PMSF) and centrifuged at 14,000 x g for 30 min at 4°C. Aliquots of supernatant were stored at -80°C. Protein concentrations in the homogenate were determined by the method of Lowry [12].

Muscle β-F1-ATPase was purified from the whole muscle homogenate by immunoprecipitation (IP) using a mouse monoclonal β-specific antibody coupled to protein G agarose beads for 1 hr at room temperature. After antibody-bead conjugation, 2 mg of muscle protein and adequate homogenization buffer was added to the mixture which was incubated overnight on rotation in 4°C. The beads were subsequently washed four times with ice-cold PBS (pH 7.4) and the proteins were denatured and eluted from the beads by incubation for 30 min at 37°C in 15 µL 2X SDS sample loading buffer [13]. Proteins were then separated by 10% SDS-polyacrylamide gel electrophoresis (PAGE) and visualized by Coomassie blue staining (Sigma Chemical Co., St. Louis, MO).

The band corresponding to the weight of β-F1-ATPase ( = 56 kD) was excised from the gel and cut into 1 mm cubes. Cubes from each lane were placed into microcentrifuge tubes and washed with 400 uL of deionized water. Coomassie stain was removed with 2 washes with 300 uL of 50% acetonitrile (ACN) in 40 mM NH_4_HCO_3_ and the gel pieces were dehydrated with 100% ACN for 15 minutes. The ACN was removed and the gel pieces were further dried in a vacuum centrifuge at 62°C for 30 min. Peptides were released from the gel by trypsin digestion (250 ng) in 30 µl of 40 mM NH_4_HCO_3_ and the samples maintained at 4°C for 15 minutes. 50 µL of 40 mM NH_4_HCO_3_ was added and the digestion was left to continue at 37°C overnight. The digestion was terminated with 10 µl of 5% formic acid (FA). Following overnight digestion, the samples were incubated at 37°C for 30 min and centrifuged for 1 min; the supernatant was transferred to a clean polypropylene tube. Another 80 µL of 0.5% FA was added to the gel pieces and the extraction procedure was repeated. Resulting peptide mixtures were purified using solid-phase extraction (C18 ZipTip; Millopore) after sample loading in 0.05% heptafluorobutyric acid:5% FA and elution with 4 uL 50% ACN:1% FA and 4 uL 80% ACN:1% FA, respectively. Eluates were combined and the sample volume was reduced to ∼2 µL by vacuum centrifugation. Subsequently, 20 µl of 0.05% heptafluorobutyric acid/1% FA:2%ACN was added as loading buffer.

### HPLC-ESI-MS/MS analysis

A hybrid mass spectrometer consisting of a Linear Ion Trap Mass spectrometer, LTQ, combined with a Fourier Transform Ion Cyclotron Resonance (FT-ICR) mass spectrometer (LTQ FT Ultra, Thermo Fisher; San Jose, CA) fitted with a PicoView nanospray source (New Objective, Woburn, MA) was used to perform the HPLC-ESI-MS/MS analyses. HPLC separations were accomplished with a linear gradient as described previously [14]. Labeled and unlabeled fragment ions of β-F1-ATPase peptides were quantified in the LTQ analyzer of the LTQ-FT-ICR instrument.

Initially, a full-scan spectrum was acquired followed by collision-induced dissociation mass spectra of the 10 most abundant ions in the survey scan to determine if there were β-F1-ATPase peptides with a high MS signal and a leucine residue in the sequence. No labeled-leucine peptides were detected in a data-dependent analysis, probably as a result of low abundance of stable-isotope-leucine enriched peptides in β-F1-ATPase. Since, any peptide with leucine has the potential to contain labeled-leucine, we therefore predicted that low abundance leucine-labeled peptides can be detected using a “targeted” mass spectrometry scan approach, and based on the addition of the appropriate mass to the mass of each leucine in a detected β-F1-ATPase peptide. A target list with various potential leucine-containing peptide m/z values was employed, resulting in improved efficiency in identifying stable-isotope-leucine enriched peptides.

Tandem mass spectra were extracted from Xcalibur “RAW” files and charge states were assigned using the Extract_MSN script which is a component of Xcalibur 2.0 SR2 (Thermo Fisher; San Jose, CA). The fragment mass spectra were searched against the IPI_RAT_v3.33 database (http://www.ebi.ac.uk/IPI/) using Mascot (Matrix Science, London, United Kingdom, version 2.2). The search parameters for the sample's protein peptides were as follows: 10 ppm mass tolerance for precursor ion masses and 0.5 Da for product ion masses; digestion with trypsin; a maximum of two missed tryptic cleavages; variable modifications of oxidation of methionine and phosphorylation of serine, threonine, and tyrosine, and addition of D9 or D10 labeled-leucine. Probability assessment of peptide assignments and protein identifications were made through use of Scaffold (version Scaffold-01_06_17, Proteome Software Inc., Portland, OR).

### β-F1-ATPase peptides confirmation and calculation of enrichment based on b and y fragment ion analysis

Peptides released from the trypsin digestion were considered only if they met the following criteria: there is a leucine residue in the amino acid sequence; detected with high intensity relative to other peptides (top 10 peptides); assigned with >95% probability to β-F1-ATPase (assessed through Scaffold Proteome Software); there is no missed cleavage; and there is no methionine in the sequence (because of methionine oxidation). There were three β-F1-ATPase unique peptides (i.e. their amino acid sequence matches only the β-F1-ATPase protein) that met all these criteria: β-F1-ATPase_95-109_ (LVLEVAQHLGESTVR; MH^+1^, mono: 1650.9), β-F1-ATPase_134-143_ (IPVGPETLGR; MH^+1^, mono: 1038.6), and β-F1-ATPase_282-294_ (VALTGLTVAEYFR; MH^+1^, mono: 1439.8). The MS/MS peak areas of the following fragment ions from these peptides were quantified (+1 charge state): β-F1-ATPase_95-109_ – b8 (890.5), b9 (1003.6), b11 (1189.7); β-F1-ATPase_134-143_ – y6 (672.4), y7 (729.4) and y8 (828.5); β-F1-ATPase_282-294_ – b8 (755.5), b9 (826.5), b10 (955.5), b11 (1118.6). The corresponding fragment ions MS/MS peak areas with +9 Da mass, containing d9-leucine, from the same peptides were also quantified. Extracted ion chromatographic peak areas corresponding to d9-labeled and unlabeled fragment ions for the three targeted β-F1-ATPase peptides were generated for the quantification of the amounts of d9-labeled and unlabeled fragments ions of the peptides in the β-F1-ATPase samples. Mass tolerance was set at 0.5 Da.

### Signal linearity in the detection of labeled and unlabeled fragment ions

Following procedures described above, a muscle homogenate was used to create four β-F1-ATPase IP samples, each containing ∼2 mg of total muscle protein. Each of the four samples was separated by SDS-PAGE and each band corresponding to the β-F1-ATPase was cut and trypsin digested. Following trypsin digestion, and after the peptide mixtures were purified using solid-phase extraction, the peptide mixtures were combined to create one sample containing an amount of β-F1-ATPase corresponding to ∼8 mg of muscle protein. This sample was dried, brought to a volume of 30 uL with loading buffer and injected into the mass spectrometer at seven different volumes corresponding to 0.2, 0.3, 0.4, 0.5, 0.8, 1.1, and 2.3 mg of muscle protein. The specific peak areas for d9-leucine-labeled and unlabeled fragment ions indicated above for the β-F1-ATPase_95-109_, β-F1-ATPase_134-143_, and β-F1-ATPase_282-294_ peptides were quantified.

### Reproducibility of the measured β-F1-ATPase enrichment

Muscle homogenate corresponding to ∼2 mg of total muscle protein was used to perform three separate β-F1-ATPase IP analyses. Each of the three samples containing immunoprecipitated β-F1-ATPase was loaded on a separate SDS-PAGE gel lane, and each band was treated as a separate sample. The three samples were analyzed in separate HPLC-MS/MS runs, using the same procedures in all three runs. The specific peak areas for d9-leucine-labeled and unlabeled fragment ions for the β-F1-ATPase_95-109_, β-F1-ATPase_134-143_, and β-F1-ATPase_282-294_ peptides were quantified. The peptide enrichment was calculated by dividing the sum of the peak areas of the labeled fragment ions by the sum of the peak areas of the corresponding unlabeled fragment ions.

### Linearity in the measured enrichment using synthetic peptides

To evaluate the linearity in the measured enrichment of the IPVGPETLGR peptide (i.e. β-F1-ATPase_134-143_), stock solutions containing leucine-labeled and unlabeled IPVGPETLGR synthetic peptides were prepared. Given the lack of commercially available d9-leucine, d10-leucine was used to synthesize the leucine-labeled peptide. D10-leucine-labeled and unlabeled IPVGPETLGR peptides were synthesized by the Proteomics and Protein Chemistry Lab facility at Arizona State University, and dissolved in water. MALDI and LC-MS/MS mass spectrometry analyses confirmed that the synthesized peptides corresponded to unlabeled and d10-leucine-labeled IPVGPETLGR peptides (Figure S1). Stock solution containing d10-labeled peptide was serially diluted with stock solution containing the unlabeled peptide to create a series of eight samples spanning a wide range of molecular ratios of labeled to unlabeled peptide. Following solid-phase extraction, and vacuum centrifugation (procedures described above), loading buffer was added to the samples prior to their injection into the mass spectrometer. Peak areas for d10-leucine-labeled and unlabeled y6-y8 fragment ions for the IPVGPETLGR peptide were quantified. These values were used to determine the peptide enrichment, and by dividing the sum of the peak areas of the labeled fragment ions by the sum of the peak areas of the corresponding unlabeled fragment ions. The molecular content of the d10-leucine-labeled and unlabeled stock solutions were calculated based on the weighted amounts of the leucine-labeled and unlabeled IPVGPETLGR peptides added to these solutions. Given the lack of purity in the synthesized peptides used in the preparation of the stock solutions, the calculated enrichment of the IPVGPETLGR peptide with d10-leucine-containing IPVGPETLGR peptide in the resultant serial dilutions represents predicted enrichment.

### Statistical analyses

Coefficient of variation (CV) values were calculated to assess the reproducibility of the enrichment measurements of the β-F1-ATPase peptides. Regression analysis was conducted to assess the linear fit between variables of interest. All the statistical analyses were performed using the Minitab® 15.1 statistical software (Minitab Inc., State College, PA).

## Results

**Figure 1:**
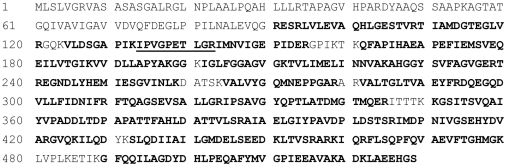
Coverage map of tryptic peptides of β-F1-ATPase from rat muscle. The muscle was analyzed using the procedures described in the text. Detected peptides are depicted in bold font letters. The amino acid sequence underlined identifies the representative tryptic β-F1-ATPase_134-143_ peptide (i.e. IPVGPETLGR). Percent coverage is 77%.

**Figure 2:**
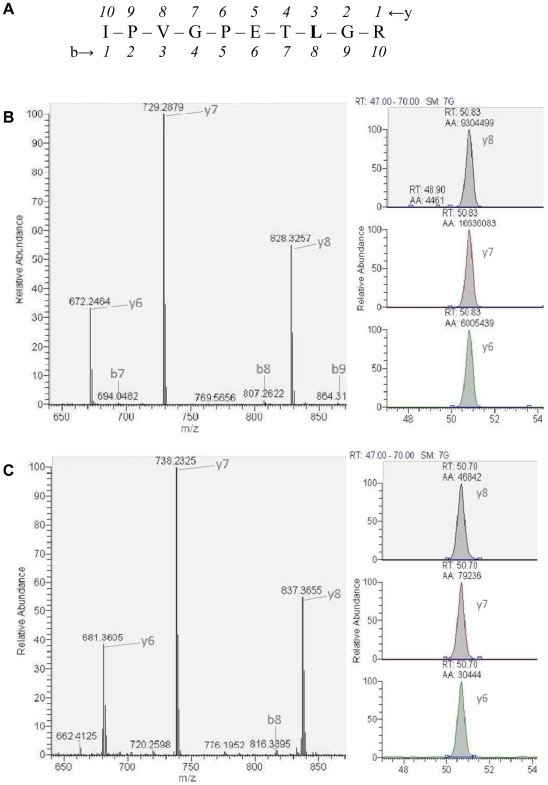
Representative IPVGPETLGR peptide of muscle β-F1-ATPase and LC-MS/MS spectra for IPVGPETLGR peptide enriched with d9-leucine. Tryptic peptide IPVGPETLGR, corresponding to β-F1-ATPase_134-143_, showing the b and y fragment ions (A); depiction of tandem mass spectrum for fragment ions of the IPVGPETLGR peptide showing the y6-y8 unlabeled fragment ions (left) and the corresponding extracted ion chromatographic peak areas of these ions (right) used for the quantification of the enrichment of the IPVGPETLGR peptide (B); depiction of spectra for fragment ions of the IPVGPETLGR peptide showing the d9-leucine-labeled fragment ions (i.e. same fragment ions as in (B) shifted by 9 m/z) (left) and the corresponding extracted ion chromatographic peak areas of these ions (right) used for quantification of the enrichment of the IPVGPETLGR peptide (C). Fragment ions either not containing leucine (i.e. b7) or containing leucine (i.e. b8, b9) in the sequence are also shown (B). Such ions, although they contain leucine and their enrichment can theoretically be quantified (i.e. b8), were not taken into consideration because their relative low abundance (<10% of parent ion).

Sequence coverage of immunoprecipitated β-F1-ATPase protein by top-10 data dependent tandem mass spectrometry following these procedures was >70% (Figure 1). Figure 2a illustrates the b and y fragment ions of the representative β-F1-ATPase_134-143_ peptide. Figure 2b-c depicts MS/MS scans for unlabeled and d9-leucine labeled y6-y8 fragment ions of the β-F1-ATPase_134-143_ peptide, together with the corresponding extracted ion chromatographic peak areas from muscle sample collected from the rat infused with the stable-isotope-labeled leucine. No peaks were detected for fragment ions with +9 Da mass in muscle β-F1-ATPase samples from the saline infused rat for any of the three targeted β-F1-ATPase peptides.

### Signal linearity in the detection of labeled and unlabeled fragment ions

**Figure 3:**
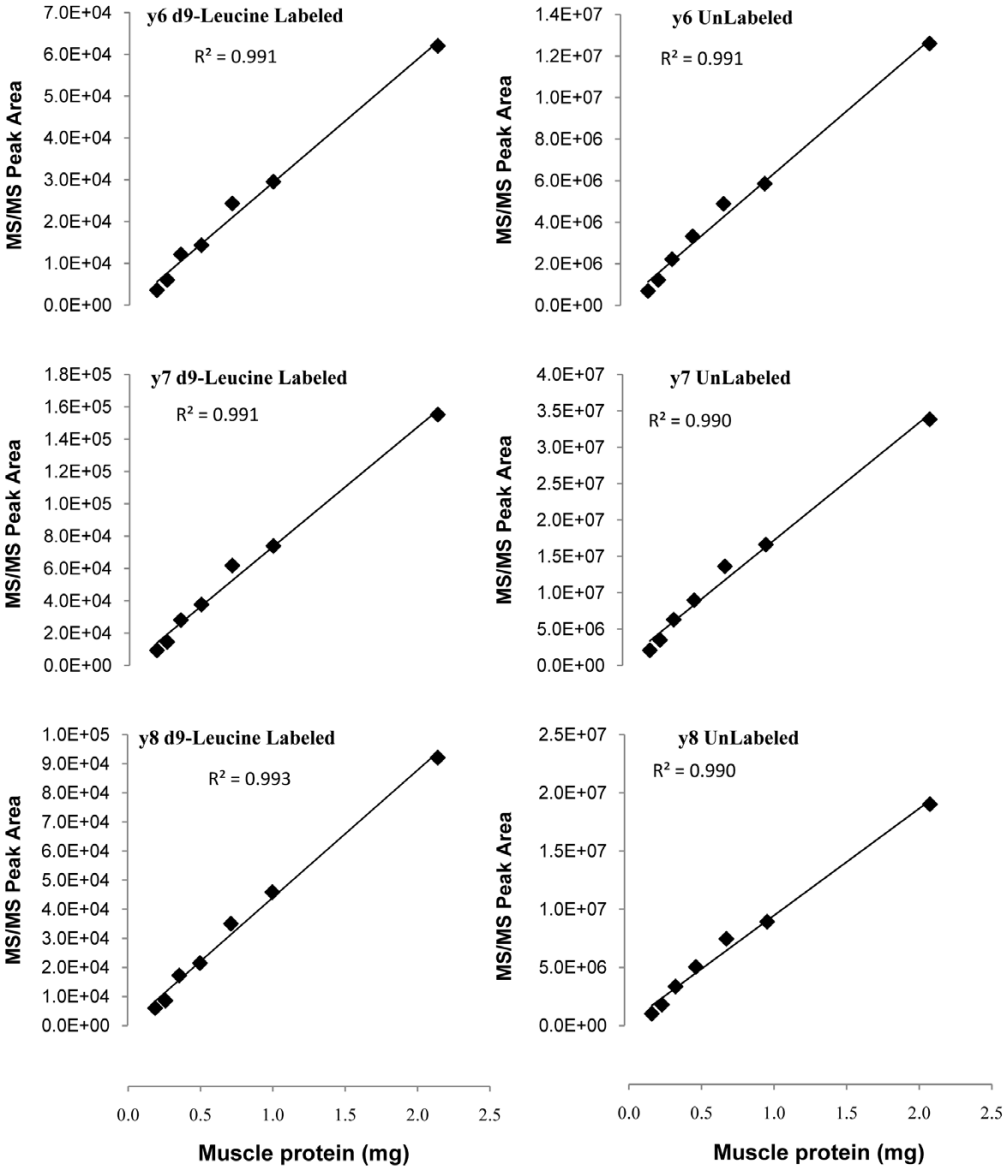
β-F1-ATPase_134-143_ peptide fragment ions peak areas as a function of the amount of protein analyzed. D9-leucine-labeled fragment ions (left) and corresponding unlabeled fragment ions (right) were quantified in association with various amounts of muscle protein processed. Data were analyzed using regression analysis. Corresponding R^2^ values are shown for each fragment ion. The y-intercept of the regression equations is not significantly different from zero (P>0.05).

For muscle β-F1-ATPase samples collected from the rat infused with the stable-isotope-labeled leucine, the MS/MS peak areas corresponding to the d9-labeled and unlabeled fragment ions of the three targeted β-F1-ATPase peptides were quantified over a wide range of amounts of protein/peptides injected into the LC-MS/MS. Regression analysis was performed between the amounts of protein analyzed, reflecting the amounts of muscle β-F1-ATPase, and each of the d9-leucine-labeled and unlabeled fragment ion peak areas of the three targeted β-F1-ATPase peptides. Calculated R^2^ values from the regression analyses for the β-F1-ATPase_95-109_ peptide corresponding to d9-labeled MS/MS peak areas ranged between 0.94 and 0.97, and those corresponding to unlabeled MS/MS peak areas ranged between 0.97 and 0.98. Regression analyses R^2^ values for the β-F1-ATPase_282-294_ peptide were between 0.62 and 0.87 for the d9-labeled MS/MS peak areas, and between 0.71 and 0.75 for the unlabeled MS/MS peak areas. R^2^ values for the ATPase_134-143_ peptide were 0.99 across all d9-labeled as well as unlabeled fragment ions (Figure 3). The regression analyses also indicated that for both the β-F1-ATPase_95-109_ and β-F1-ATPase_282-294_ peptides the y-intercept of the regression equations describing the relationship between the amounts of protein analyzed and fragment ion peak areas were significantly different from zero (*P*<0.05) for all d9-labeled and unlabeled fragment ions. However, none of the y-intercept values of the regression equations describing the corresponding relationships for the ATPase_134-143_ peptide were significantly different from zero (*P*>0.05).

### Reproducibility of the measured β-F1-ATPase enrichment

For each β-F1-ATPase peptide within a replicate the sum of the peak areas of its d9-leucine-labeled fragment ions was divided by the sum of the peak areas of its corresponding unlabeled fragment ions in order to determine the enrichment of the specific β-F1-ATPase peptide with d9-leucine. The average percent enrichment based on these replicates for the β-F1-ATPase_95-109_ peptide was 0.564% ± 0.023% (average value ± standard deviation), with a CV value of 4.15%. These same measures corresponding to the β-F1-ATPase_134-143_ peptide were 0.482% ± 0.014% and 2.6%, respectively. For the β-F1-ATPase_282-294_ peptide the average percent enrichment was 0.566% ± 0.051% and the CV was 8.98%.

### Linearity in the measured enrichment using synthetic peptides

**Figure 4:**
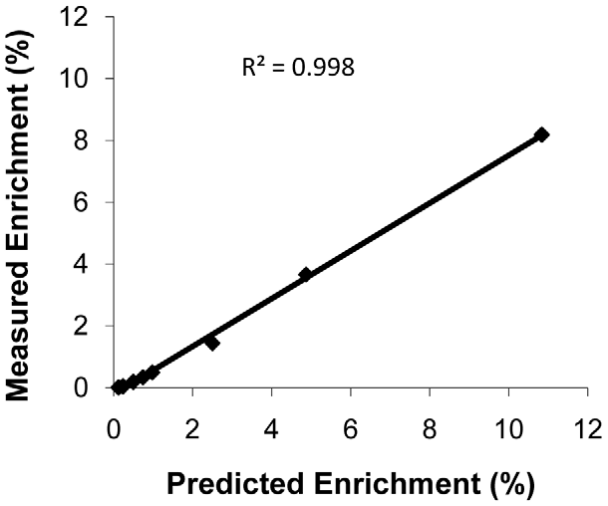
Relationship between measured and predicted enrichment for the synthetic peptide IPVGPETLGR. D10-leucine labeled and unlabeled IPVGPETLGR synthetic peptides were mixed at eight different molar ratios, processed, and analyzed by LC-MS/MS. The peak areas for d10-leucine-labeled and unlabeled y6-y8 fragment ions for the IPVGPETLGR peptide were quantified. Peptide percent enrichment was calculated by dividing the sum of the peak areas of the labeled fragment ions by the sum of the peak areas of the corresponding unlabeled fragment ions. Data between measured and predicted percent enrichments were analyzed using regression analysis.

Linearity in the measured enrichment was evaluated only for the β-F1-ATPase_134-143_ peptide (i.e. IPVGPETLGR), because it was the only peptide that showed acceptable linearity in the signal intensity in the detection of labeled and unlabeled fragment ions (based on the results reported in the “Signal Linearity in the Detection of Labeled and Unlabeled Fragment Ions” section). The measured percent enrichment (i.e. sum of y6-y8 labeled fragment ions peak areas/sum of y6-y8 unlabeled fragment ions peak areas*100) in the LC-MS/MS-analyzed samples prepared to contain synthetic IPVGPETLGR peptides enriched with synthetic IPVGPETLGR peptides that contained labeled leucine ranged between 0.009% and 8.185%. Regression analysis indicated excellent linear relationship between the measured and predicted IPVGPETLGR percent enrichment throughout the range of the prepared samples (Figure 4).

## Discussion

The overall goal of this study was to evaluate the reproducibility of an LC-MS/MS approach to quantify the enrichment of *in vivo*-labeled muscle β-F1-ATPase based on the enrichment of its tryptic peptides. Measurement of this enrichment is a key methodological step in order to evaluate the translational regulation of β-F1-ATPase by means of stable-isotope methodology. The described approach is based on immunoprecipitation of β-F1-ATPase, separation of β-F1-ATPase by 1-D SDS-PAGE, and determination of the enrichment of fragment ions of selected β-F1-ATPase peptides using LC-MS/MS.

An important finding of the present study is that two (i.e. β-F1-ATPase_95-109_ and β-F1-ATPase_282-294_) of the three tryptic β-F1-ATPase peptides that met our initial criteria to be used for the determination of the reproducibility of β-F1-ATPase peptide enrichment demonstrated low, and in some cases unacceptable (i.e. R^2^ = 0.62), linearity in the signal intensity with which amounts of labeled and unlabeled fragment ions were detected. For these two peptides, there was proportionally less increase in MS/MS fragment ion signal intensity with increased amounts of protein/peptides injected into the LC-MS/MS. On the other hand, there was an excellent linearity (i.e. R^2^ = 0.99) in the signal intensity with which labeled and unlabeled amounts of fragment ions were detected for the β-F1-ATPase_134-143_ peptide. Also, none of the y-intercept values of the regression equations describing the MS/MS fragment ion intensities as a function of the amounts of protein/peptide injected into the LC-MS/MS was significantly different from zero for the β-F1-ATPase_134-143_ peptide. The latter finding further underscores an accurate detection of generated amounts of labeled and unlabeled fragment ions for the β-F1-ATPase_134-143_ peptide over a wide range of labeled and unlabeled amounts of protein analyzed. Although the specific reason(s) for the lack of linear function in the signal intensity with which labeled and unlabeled amounts of fragment ions were detected for the β-F1-ATPase_95-109_ and β-F1-ATPase_282-294_ peptides are not known, visual inspection of their MS/MS spectra showed poor quality spectra in association with greater noise when compared to the MS/MS spectra of the β-F1-ATPase_134-143_ peptide.

The results obtained with respect to the percent enrichment of the β-F1-ATPase_134-143_ peptide in the reproducibility experiments (“Reproducibility of the Measured β-F1-ATPase Enrichment” section) are similar to those when the percent enrichment (0.470% ± 0.012%) and CV (2.6%) for the β-F1-ATPase_134-143_ peptide were calculated based on the data obtained across the various amounts of the protein/peptide analyzed in the MS/MS signal linearity experiment (“Signal Linearity in the Detection of Labeled and Unlabeled Fragment Ions” section). The latter findings enhance the results obtained from the reproducibility experiments for the β-F1-ATPase _134-143_ peptide and provide additional evidence for an excellent reproducibility in the measurement of *in vivo*-labeled muscle β-F1-ATPase enrichment using LC-MS/MS when this measurement is based on the enrichment of the β-F1-ATPase _134-143_ peptide. The presence of more than one leucine residues that can be labeled with d9-leucine in both the β-F1-ATPase_95-109_ and β-F1-ATPase_282-294_ peptides could be the reason for the poorer reproducibility of the enrichment measurement using those two peptides. There was also greater average percent enrichment measured using the β-F1-ATPase_95-109_ and the β-F1-ATPase_282-294_ peptides compared to that measured using the β-F1-ATPase _134-143_ peptide that is attributed to the existence of isobaric peptides for the β-F1-ATPase_95-109_ and the β-F1-ATPase_282-294_. The probability of having a singly labeled leucine in any given peptide increases with the number of amino acid (i.e. leucine) residues that can incorporate labeled leucine, and the β-F1-ATPase_95-109_ and the β-F1-ATPase_282-294_ peptides both contain more than one leucine residues in their sequence.

Previously published approaches to measure the enrichment of proteins labeled *in vivo* with stable isotopes in animal models [15] were done in conjunction with high isotopic enrichment of the proteins (i.e. feeding the animals highly enriched isotopes for very long periods of time), none of which is practical in short-term (i.e. only hours-long) animal or human experiments. Also, contrary to the circumstance related to the determination of the enrichment of a protein with a fast turnover rate such as the amyloid-β in human cerebrospinal fluid [9], the slow turnover rate, and thus the lower abundance of labeled β-F1-ATPase molecules in muscle, constitutes a challenge to reproducibly measure the stable-isotope enrichment of muscle β-F1-ATPase, and in a way that is practical when investigating its kinetics in animals or humans. The present study builds on the previously described approach [9], and extents the methodology as well as its applicability to a muscle protein. Evaluation of the reproducibility of the measurement of the stable-isotope enrichment of peptides of a specific muscle protein using LC-MS/MS is prerequisite for the use of any peptide-based approach that is employed to measure the kinetics of such protein in association with stable-isotope infusion experiments in animals or humans. Our results show that the stable-isotope enrichment of *in-vivo*-labeled muscle β-F1-ATPase can reliably be measured based on the determination of the enrichment of the β-F1-ATPase_134-143_ peptide.

Recently Jaleel et al. [16], have reported an MS/MS approach to determine the stable-isotope enrichment of muscle proteins labeled *in vivo*. This approach involves two separate steps, with the first being the identification of a protein that is separated by two-dimensional gel electrophoresis using LC-MS/MS, and the second the measurement of the enrichment of the identified protein using GC-MS/MS. Protein enrichment in the latter step is determined by measuring the stable-isotope enrichment of an amino acid following hydrolysis of the identified protein. Besides using a single MS/MS step in the analysis of a pre-selected peptide, the approach described herein is based on the measurement of the stable-isotope enrichment of a unique peptide of the muscle protein. This is important, because when determination of protein enrichment is based on the determination of the enrichment of its amino acids the accuracy of the measured enrichment depends on the purity of the isolated protein in the sample (i.e. contamination of the amino acids of the protein of interest by hydrolyzed amino acids from unrelated proteins). A major advantage of the approach described herein is, therefore, the specificity in the determination of the enrichment of the protein of interest.

It is noted that the stable-isotope of the amino acid leucine evaluated in the present study contains nine deuterium atoms, which ensures practically zero d9-leucine β-F1-ATPase enrichment at the initiation of the infusion period. Therefore, a single muscle biopsy performed at the end of the period of interest [17], [18], [19] in combination with frequent peripheral blood sampling can be used to accurately describe the rate of synthesis of β-F1-ATPase. Because the same peptide (IPVGPETLGR) is observed in muscle from both rats (β-F1-ATPase_134-143_) and humans (β-F1-ATPase_134-143_) [13], measurement of β-F1-ATPase enrichment based on the enrichment of this peptide is readily applicable to human experiments. Although we have specifically been interested in β-F1-ATPase, this approach can be applied in the determination of the enrichment of any protein from skeletal muscle that has been labeled in vivo, and after evaluating the existence of a peptide that can reliably be used to describe the enrichment of the protein of interest. With respect to the applicability of the approach described herein only the abundance of the protein of interest in the sample is a concern. In the present study, 2 mg of muscle protein (i.e. 20 mg of wet muscle) are sufficient for the determination of β-F1-ATPase enrichment. However, a larger muscle sample can increase the overall amount of labeled proteins found in smaller quantities in skeletal muscle. In this regard, and although in the present studies mitochondrial β-F1-ATPase was immunoprecipitated from whole muscle homogenate, isolation of the mitochondria can enrich the protein homogenate with mitochondrial proteins that are targeted for quantitative determination several fold [20].

In conclusion, our results show that the reproducibility of the determination of the stable-isotope enrichment of a protein using LC-MS/MS may vary based on the specific peptide chosen to describe the protein enrichment. The β-F1-ATPase_134-143_ peptide provides reproducible results for the determination of the stable-isotope enrichment of *in vivo*-labeled muscle β-F1-ATPase. Although we have been interested in the enrichment of β-F1-ATPase, this approach can be extended in the study of numerous other proteins from skeletal muscle.

## Supporting Information

Figure S1 **MALDI and LC-MS/MS mass spectrometry analysis of synthetic peptides.** Synthetic IPVGPETLGR peptides, corresponding to the tryptic peptide of the β-F1-ATPase (i.e. β-F1-ATPase), were prepared to contain unlabeled and d10-labeled-leucine. The top part shows MALDI spectra for the unlabeled synthetic peptide (A) and the labeled-leucine synthetic peptide (B), the latter shifted by 10 Da. The bottom part shows LC-MS/MS spectra for fragment ions for the unlabeled synthetic peptide (C) and the labeled-leucine synthetic peptide (D), the latter shifted by 10 Da. These mass spectrometry data provide evidence that the synthesized peptides correspond to unlabeled and d10-leucine-labeled IPVGPETLGR peptides.
(TIF)
