# Supplementary figures and images for: Reproducibility of an HPLC-ESI-MS/MS Method for the Measurement of Stable-Isotope Enrichment of in Vivo-Labeled Muscle ATP Synthase Beta Subunit
Source: PLoS One. 2011 Oct 12;6(10):e26171. doi: 10.1371/journal.pone.0026171 (PMC3192170; doi:10.1371/journal.pone.0026171)

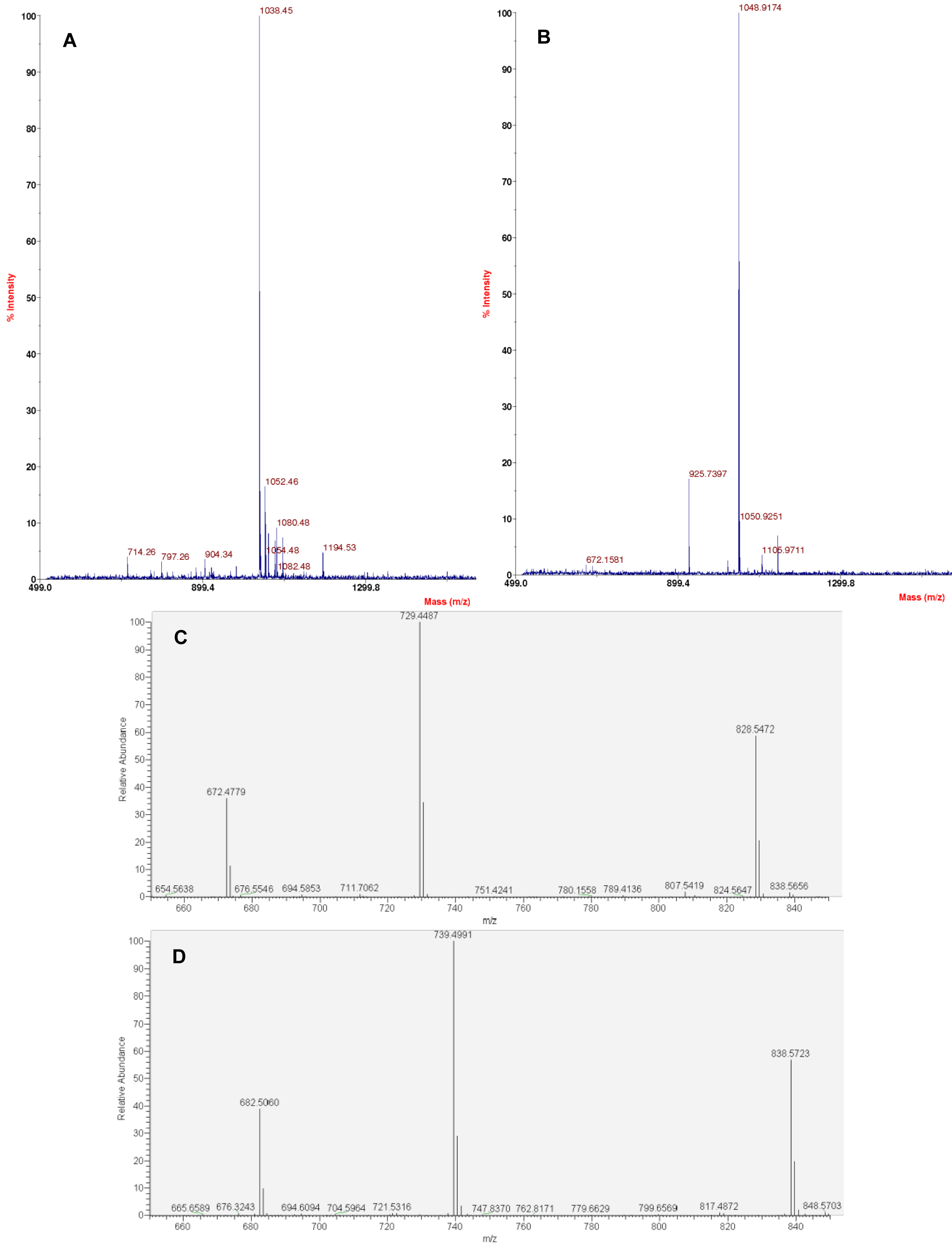

Supplement: Figure S1 — MALDI and LC-MS/MS mass spectrometry analysis of synthetic peptides. Synthetic IPVGPETLGR peptides, corresponding to the tryptic peptide of the β-F1-ATPase (i.e. β-F1-ATPase), were prepared to contain unlabeled and d10-labeled-leucine. The top part shows MALDI spectra for the unlabeled synthetic peptide (A) and the labeled-leucine synthetic peptide (B), the latter shifted by 10 Da. The bottom part shows LC-MS/MS spectra for fragment ions for the unlabeled synthetic peptide (C) and the labeled-leucine synthetic peptide (D), the latter shifted by 10 Da. These mass spectrometry data provide evidence that the synthesized peptides correspond to unlabeled and d10-leucine-labeled IPVGPETLGR peptides. (TIF) [file pone.0026171.s001.tif]
